# Platelet Adhesion and Activation in an ECMO Thrombosis‐on‐a‐Chip Model

**DOI:** 10.1002/advs.202401524

**Published:** 2024-05-17

**Authors:** Tiffany Goh, Lingzi Gao, Jasneil Singh, Richard Totaro, Ruaidhri Carey, Kevin Yang, Bruce Cartwright, Mark Dennis, Lining Arnold Ju, Anna Waterhouse

**Affiliations:** ^1^ School of Medical Sciences, Faculty of Medicine and Health The University of Sydney Sydney NSW 2006 Australia; ^2^ Heart Research Institute Newtown NSW 2042 Australia; ^3^ Charles Perkins Centre The University of Sydney Sydney NSW 2006 Australia; ^4^ The University of Sydney Nano Institute The University of Sydney Sydney NSW 2006 Australia; ^5^ Faculty of Medicine and Health The University of Sydney Sydney NSW 2006 Australia; ^6^ Intensive Care Department Royal Prince Alfred Hospital Missenden Road, Camperdown Sydney NSW 2050 Australia; ^7^ Anaesthetics Department Royal Prince Alfred Hospital Camperdown Sydney NSW 2050 Australia; ^8^ Cardiology Department Royal Prince Alfred Hospital Missenden Road, Camperdown Sydney NSW 2050 Australia; ^9^ School of Biomedical Engineering Faculty of Engineering The University of Sydney Darlington NSW 2008 Australia

**Keywords:** computational fluid dynamics, extracorporeal circuits, microfluidics, platelets, thrombosis

## Abstract

Use of extracorporeal membrane oxygenation (ECMO) for cardiorespiratory failure remains complicated by blood clot formation (thrombosis), triggered by biomaterial surfaces and flow conditions. Thrombosis may result in ECMO circuit changes, cause red blood cell hemolysis, and thromboembolic events. Medical device thrombosis is potentiated by the interplay between biomaterial properties, hemodynamic flow conditions and patient pathology, however, the contribution and importance of these factors are poorly understood because many in vitro models lack the capability to customize material and flow conditions to investigate thrombosis under clinically relevant medical device conditions. Therefore, an ECMO thrombosis‐on‐a‐chip model is developed that enables highly customizable biomaterial and flow combinations to evaluate ECMO thrombosis in real‐time with low blood volume. It is observed that low flow rates, decelerating conditions, and flow stasis significantly increased platelet adhesion, correlating with clinical thrombus formation. For the first time, it is found that tubing material, polyvinyl chloride, caused increased platelet P‐selectin activation compared to connector material, polycarbonate. This ECMO thrombosis‐on‐a‐chip model can be used to guide ECMO operation, inform medical device design, investigate embolism, occlusion and platelet activation mechanisms, and develop anti‐thrombotic biomaterials to ultimately reduce medical device thrombosis, anti‐thrombotic drug use and therefore bleeding complications, leading to safer blood‐contacting medical devices.

## Graphical abstract

Based on clinical ECMO flow and thrombosis location, an ECMO thrombosis‐on‐a‐chip model is developed with customizable biomaterial and flow combinations, which uses low blood volumes. Real‐time microscopy revealed that low flow rates, deceleration, and stasis significantly increases acute platelet adhesion, and occlusion and embolism can be visualized. Increased platelet activation is observed on polyvinyl chloride compared to polycarbonate.

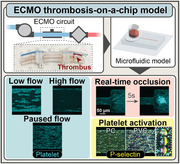

## 1 Introduction

Extracorporeal membrane oxygenation (ECMO) provides end‐organ perfusion support for patients with severe cardiac and/or respiratory failure,^[^ ^1^, ^2^ ^]^ however, blood exposure to the ECMO circuit's foreign biomaterials and pathological flow conditions initiates dangerous blood clot formation (thrombosis).^[^ ^3^, ^4^ ^]^ Thrombotic events (e.g., circuit component thrombosis, oxygenator failure, hemolysis, venous thromboembolism and ischemic stroke) occur in 17 – 35% of circuits^[^ ^5^, ^6^, ^7^ ^]^ and affect patient survival (60% in veno‐venous (VV) ECMO^[^ ^8^ ^]^). This is despite the use of anticoagulants (e.g., unfractionated heparin or direct thrombin inhibitors such as bivalirudin and argatroban),^[^ ^2^, ^9^ ^]^ as well as heparin‐ and albumin‐coated circuits,^[^ ^5^, ^10^ ^]^ last reported to be used in 59% of centers,^[^ ^11^ ^]^ which together aim to reduce device thrombosis. Furthermore, anticoagulation increases the risk of bleeding complications (e.g., surgical site bleeding, intracranial hemorrhage and gastrointestinal bleeding) which occur in approximately 30 – 60% of patients^[^ ^6^, ^12^, ^13^ ^]^ and also affect patient survival. Overall, circuit component thrombosis and hemofilter clots accounted for 4.4, 12.8, and 23.2% of reported complications in adult, paediatric and neonate patients, respectively.^[^ ^14^ ^]^

The three key factors that cause medical device thrombus formation and influence clot properties are summarised by a modified Virchow's Triad: patient pathology, material surface properties and blood flow hemodynamics.^[^ ^15^, ^16^, ^17^ ^]^ Flow hemodynamics are especially important in ECMO thrombosis as the circuit configuration (flow rate, tubing connectors, flow transitions to and from circuit components, and circuit stopping during treatment), patient physiological requirements, and underlying pathology result in different flow regimes for each patient. As a result, blood is exposed to various flow velocities, acceleration, deceleration and shear rates (the force due to fluid flow on blood cells and proteins, measured in s^−1^).^[^ ^15^ ^]^ The interplay between these flow conditions and the material properties that drive thrombosis is incompletely understood, limiting our ability to reduce ECMO thrombosis and develop non‐thrombogenic materials for ECMO. A major reason for this is that current in vitro models of biomaterial thrombosis rarely take these ECMO‐specific flow regimes into account, lacking the ability to recapitulate high‐ and low‐shear flow and shear gradients, limiting the field's knowledge of biomaterial thrombosis at clinically relevant ECMO flow conditions.

This research presents a novel ECMO thrombosis‐on‐a‐chip model with tailorable and clinically relevant flow, biomaterial, and blood selection for real‐time analysis of ECMO thrombosis. We evaluated the adhesion and activation of platelets to indicate the initiation of thrombosis in microfluidics using real‐time confocal microscopy, mimicking ECMO tubing and tubing‐connector junction (TCJ) shear rates and biomaterials. We found that low shear rates, regions of decelerating flow, and stopping the blood flow pump significantly increased platelet adhesion. Meanwhile, although no difference in total platelet adhesion was observed on polyvinyl chloride (PVC) compared to polycarbonate (PC), increased expression of the platelet activation marker P‐selectin was observed on PVC compared to PC, demonstrating the importance of the material in activation of thrombosis. The results from this model can be used to guide clinical ECMO operating conditions, inform future ECMO geometry redesign to optimize flow regimes, study mechanisms of platelet mediated biomaterial thrombosis and develop new anti‐thrombotic materials for ECMO and other blood‐contacting medical devices. The ultimate aim of this model is to contribute to reducing medical device thrombosis and the use of anticoagulants and antiplatelet drugs clinically to prevent patient bleeding complications.

## 2 Results

### 2.1 Replicating ECMO Tubing Thrombosis Conditions on‐a‐Chip

**Figure 1:**
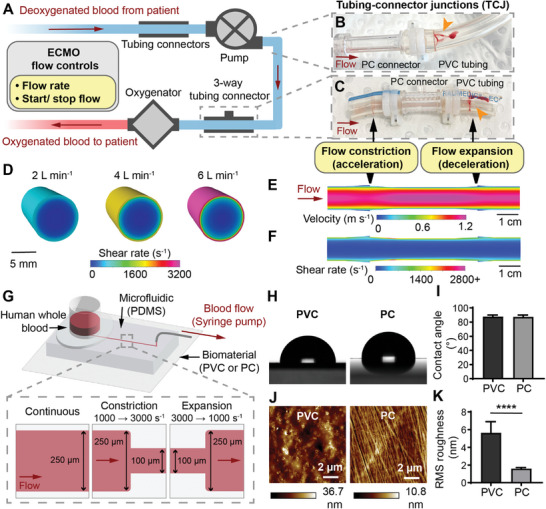
Replicating ECMO tubing thrombosis conditions on‐a‐chip. A) Schematic of ECMO circuit configuration and flow controls. B) ECMO pump outlet connector to tubing junction, showing thrombus (orange arrowhead). C) ECMO 3‐way TCJ showing thrombus (orange arrowhead). D) CFD model of the wall shear rate in ECMO tubing at flowrates of 2, 4 and 6 L min^−1^. CFD model of the E) velocity and F) shear rate, in an ECMO TCJ at 4 L min^−1^. G) Schematic of the ECMO thrombosis‐on‐a‐chip microfluidic model, and channel dimensions for different flow regimes. H) Representative photographs of the water contact angle on PVC and PC. I) Water contact angle of PVC and PC showed no significant difference (*n* = 9 droplets). J) Representative atomic force micrographs of PVC and PC. K) The RMS roughness of PVC was significantly greater than PC (*n* = 9 scans). Error bars are mean ± SD. Significance comparisons are *P < 0.05, **P < 0.01, ***P < 0.001, ****P < 0.0001 by an unpaired Student's *t*‐test.

ECMO circuits consist of plasticized polyvinyl chloride (PVC) tubing (inner diameter I.D. 3/8**′′**) that transports blood between the patient, blood pump and oxygenator (**Figure** **1**A). Polycarbonate connectors (outer diameter O.D. 3/8**′′**) interconnect the pump, oxygenator, and sections of tubing (Figure 1B,C). The tubing‐connector junctions (TCJs) from a patient on veno‐venous (VV) ECMO showed visible thrombus formation (Figure 1B,C, orange arrowheads), correlating with numerous other clinical reports of them as sites of visible thrombus stabilization.^[^ ^18^, ^19^ ^]^

Computational fluid dynamics (CFD) modeling was used to obtain clinically relevant ECMO tubing and TCJ flow parameters to replicate in vitro. Adult ECMO flow rates can range from 2–6 L min^−1^, with an average of 4 L min^−1^ depending on patient‐specific weight, oxygen saturation and cardiac output requirements.^[^ ^2^ ^]^ CFD modeling of blood shear rates at the biomaterial surface at 2, 4 and 6 L min^−1^ was 571, 1654, and 3150 s^−1^ (wall shear stress of 1.97, 5.71, and 10.87 Pa) respectively in the tubing (I.D. 9.525 mm), and peaked at 888, 2699, 5128 s^−1^ (wall shear stress of 3.09, 9.31, 17.92 Pa) respectively along the smooth connector surface (I.D. 8.9 mm) (Figure 1D,F and **Table** **1**). TCJ CFD models identified that the critical region where large clots were observed in Figure 1B,C were areas of constricting or expanding flow with near stagnant velocity (Figure 1E,F). Having established ECMO‐relevant flow parameters, straight microfluidic channels (w: 250 µm, h: 100 µm) were operated at 500 – 5000 s^−1^ (calculated from ^[^ ^20^ ^]^) to model ECMO shear rates, and constricting and expanding microfluidic channels transitioning between 1000 s^–1^ (w: 250 µm) to and from 2000 (w: 135 µm), 3000 (w: 100 µm) and 5000 s^−1^ (w: 75 µm) mimicked ECMO TCJ shear rates and gradients (Figure 1G).

**Table 1:** CFD modeling results of the wall shear rate (s^−1^) and wall shear stress (Pa) at the biomaterial surface of ECMO tubing (I.D. 9.525 mm) and TCJ (I.D. 8.9 mm) at 2, 4, and 6 L min^−1^.

	Tubing [I.D. 9.525 mm]		TCJ [narrowest I.D. 8.9 mm]	
	Wall shear rate [s^−1^]	Wall shear stress [Pa]	Wall shear rate [s^−1^]	Wall shear stress [Pa]
2 L min^−1^	571	1.97	888	3.09
4 L min^−1^	1654	5.71	2699	9.31
6 L min^−1^	3150	10.87	5128	17.92

The microfluidics’ base used commercially available unplasticized PVC and PC sheets to observe blood‐biomaterial interface interactions under flow, mimicking uncoated ECMO circuits (Figure 1G). Biomaterial surface roughness and wettability are known to effect biomaterial thrombogenicity and clot properties,^[^ ^17^, ^21^ ^]^ and were evaluated using atomic force microscopy and water contact angle measurements. There was no significant difference between the wettability of PVC (87.8 ± 2.2°) and PC (87.5 ± 2.4°) (Figure 1H,I). Although the surface roughness was extremely low, PVC (5.63 ± 1.27 nm) was rougher than PC (1.6 ± 0.12 nm, *P* < 0.0001) (Figure 1J,K).

### 2.2 Increasing Continuous Shear Rate Decreased Platelet Adhesion on PVC and PC

The effect of six ECMO‐relevant flow conditions introduced in Figure 1 on platelet, red blood cell, neutrophil and monocyte adhesion, and fibrin polymerisation, were initially screened using heparinised, citrated, and recalcified citrated blood (Figure S1, Supporting Information). Heparin mimicked the clinical ECMO treatment standard where patients are anticoagulated with continuous heparin infusion, despite which thrombosis still occurs.^[^ ^9^ ^]^ We found that citrate showed non‐significantly different platelet adhesion to heparin at our selected flow conditions (Figures S1A,B and S2, Supporting Information). Therefore, citrate was used as it had the added benefits compared to heparin of a longer half‐life and provided less time‐sensitivity for in vitro testing. Recalcified citrated blood mimicked anticoagulant free blood and showed rapid fibrin polymerization, red blood cell and neutrophil adhesion (Figure S1C, Supporting Information). Although non‐anticoagulated conditions are not relevant to standard ECMO treatment, this result demonstrated the model's ability to detect coagulation and adhesion of fibrin and other blood cell types when present.

**Figure 2:**
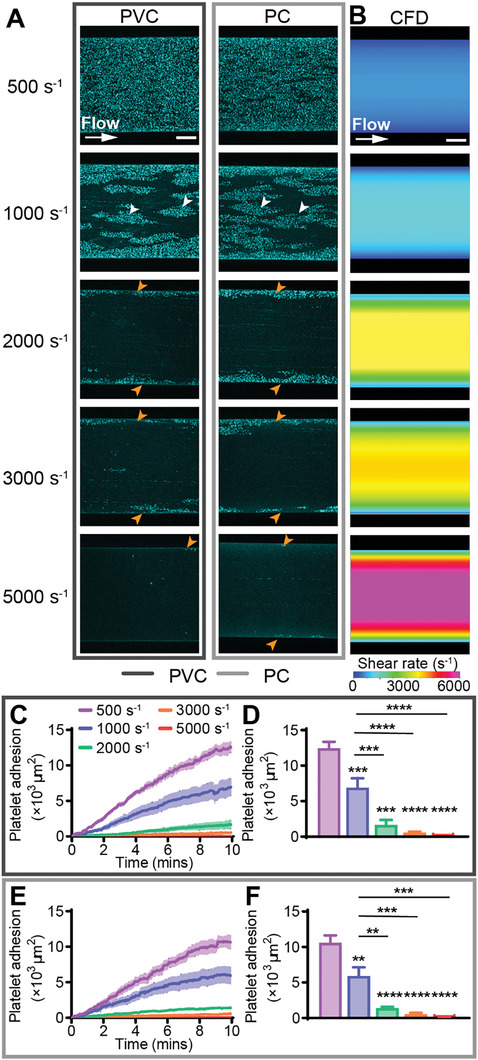
Increasing continuous shear rate decreased platelet adhesion on PVC and PC. A) Representative confocal micrographs of platelet adhesion (cyan) on PVC and PC after 10 min of continuous flow at 500, 1000, 2000, 3000, and 5000 s^−1^. Scale bar = 50 µm. B) CFD model of the shear rate at h = 2 µm (approximate platelet level) above the biomaterial surface at target shear rates of 500, 1000, 2000, 3000, and 5000 s^−1^. Scale bar = 50 µm. Total fluorescent surface area indicating platelet adhesion over 10 min on C) PVC and E) PC. Quantification of total platelet adhesion at 10 min showed a statistically significant decrease in platelet adhesion from 500 and 1000 s^−1^ to 2000, 3000, and 5000 s^−1^ on D) PVC and F) PC. Error bars are mean ± SEM, *n* = 5 donors. Significance comparisons are *P < 0.05, **P < 0.01, ***P < 0.001, ****P < 0.0001 by an ordinary one‐way ANOVA with Bonferroni's post hoc test. Significance presented compared to 500 s^−1^ unless otherwise indicated.

To determine whether acute platelet adhesion to biomaterials was dependent on ECMO flow rate and thus shear rate, citrated human whole blood was perfused over PVC and PC at 500, 1000, 2000, 3000, or 5000 s^−1^ and platelet adhesion recorded over 10 min. At 500 s^−1^, platelets were dispersed in an even monolayer across the channel on both PVC and PC, however at 1000 s^−1^ platelet deposition was only continuous at the near‐wall low shear region, with discrete large clusters of platelets at the higher‐shear region in the middle of the channel (**Figure** **2**A, white arrowheads, Movie S1, Supporting Information). As the shear rate was increased to 2000, 3000 and 5000 s^−1^, platelet adhesion was confined to the near‐wall low‐shear region only, with small clusters or discrete platelets in the mid‐channel region (Figure 2A, orange arrowheads, Movie S1, Supporting Information). Compared to CFD models of the shear rate distribution across the microfluidic channel, there was reduced platelet adhesion at areas exceeding 2000 s^−1^ (Figure 2B). No platelet aggregate, red blood cell, neutrophil and monocyte adhesion and only minimal fibrin formation were recorded for these flow conditions (Figure S1A, Supporting Information and data not shown).

On both PVC and PC, increasing the shear rate above 500 and 1000 s^−1^ significantly decreased platelet adhesion after 10 minutes. On PVC, platelet adhesion at 1000, 2000, 3000, and 5000 s^−1^ was 1.8 ± 0.5, 7.4 ± 3.6, 21.0 ± 7.0, and 311.3 ± 130.9 ‐fold less than at 500 s^−1^ respectively (all *P* < 0.001), and platelet adhesion at 2000, 3000, and 5000 s^−1^ was 4.1 ± 2.0, 11.7 ± 3.9, and 173.1 ± 72.8 ‐fold less than at 1000 s^−1^ respectively (all *P* < 0.001) (Figure 2C,D). On PC, platelet adhesion at 1000, 2000, 3000, and 5000 s^−1^ was 1.8 ± 0.6, 7.5 ± 1.6, 18.7 ± 8.0, and 168.0 ± 54.5 ‐fold less than at 500 s^−1^ respectively (all *P* < 0.01), while platelet adhesion at 2000, 3000, and 5000 s^−1^ was 4.2 ± 0.9, 10.4 ± 4.5, and 93.7 ± 30.4 ‐fold less than at 1000 s^−1^ respectively (all *P* < 0.01) (Figure 2E,F). No statistically significant differences between platelet adhesion on PVC and PC at the tested shear rates were recorded (Figure S3A, Supporting Information).

### 2.3 Accelerating Flow Regions Decreased Platelet Adhesion and Promoted Embolism

**Figure 3:**
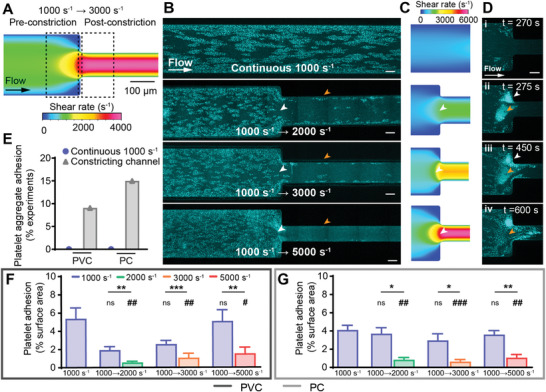
Accelerating flow regions decreased platelet adhesion and promoted embolism. A) Schematic of the 1000 to 3000 s^−1^ constricting microfluidic, with dotted boxes representing areas for statistical analysis. B) Representative confocal micrographs of platelet adhesion (cyan) on PVC after 10 min of perfusion at continuous flow, or accelerating from 1000 s^−1^ to 2000, 3000, or 5000 s^−1^. Scale bar = 50 µm. C) CFD model of the wall shear rate in tested constricting microfluidic channels. D) Representative confocal micrograph time‐course of a 1000 to 5000 s^−1^ PC microfluidic channel showing platelet aggregate adhesion and embolism over time. Scale bar = 50 µm. E) Percentage of experiments with platelet aggregate adhesion in continuous and constricting microfluidic channels on PVC (n = 0/19 and n = 2/22 respectively) and PC (n = 0/21 and n = 3/20 respectively). Analysis of total fluorescent surface area indicating platelet adhesion at 10 min on F) PVC and G) PC constricting channels. Error bars are mean ± SEM, *n* = 5 donors where platelet aggregate adhesion did not occur. Significance comparisons between pre‐ and post‐constriction platelet adhesion for each constricting channel are *P < 0.05, **P < 0.01, ***P < 0.001 by a paired Student's *t*‐test. Significance comparisons to the continuous 1000 s^−1^ channel are #*P* < 0.05, ##*P* < 0.01, ###*P* < 0.001 by an ordinary one‐way ANOVA with Bonferroni's post hoc test.

To model the inlet of TCJs, we designed constricting microfluidic channels that mimicked the shear regime where ECMO blood flows from the tubing (larger diameter) into the connector (smaller diameter) resulting in flow acceleration and increasing shear rate (Figure 1E,F and **Figure** **3**A). Continuous shear at 1000 s^−1^ was compared to three constricting‐flow shear gradients of 1000 to 2000 s^−1^, 1000 to 3000 s^−1^, and 1000 to 5000 s^−1^.

For constricting channels that did not adhere large platelet aggregates, the pre‐constriction (1000 s^−1^) region adhered platelets in a monolayer of clusters, similarly to the continuous 1000 s^−1^ channel (Figure 3B; Figure S4B, Supporting Information). However, at the constriction point, there was an area of reduced platelet adhesion (Figure 3B, white arrowhead), correlating to CFD modeling indicating an increasing shear gradient (Figure 3C, white arrowhead). Post‐constriction (2000, 3000 or 5000 s^−1^), platelet adhesion was confined to the near‐wall (approaching 0 s^–1^) regions for all three tested constriction gradients (Figure 3B, orange arrowhead, Figure S4B, Supporting Information).

Platelet aggregates were not observed in any straight (continuous 1000 s^−1^) microfluidic channels (PVC n = 0/19, PC n = 0/21 channels), but were recorded in constricting flow channels (Figure 3D) in 9.1% (n = 2/22) of PVC and 15% (n = 3/20) of PC channels (Figure 3E). In channels that did contain aggregates, the aggregates adhered both against the PDMS wall of the constriction point (Figure 3Dii, white arrowhead) or on the biomaterial sample (Figure 3Dii, orange arrowhead). Over time, the aggregates bound additional platelets, most rapidly growing at the leading end of the aggregate (Movie S2, Supporting Information). In some cases, the multilayer platelet aggregates formed an embolism and detached from the channel, but left a dense monolayer of platelets on the biomaterial surface (Figure 3Div, orange arrowhead, Movie S2, Supporting Information).

Statistical analysis of total platelet adhesion was conducted for five independent donors that did not show platelet aggregate adhesion. The percentage area containing platelet adhesion was analyzed as the percentage fluorescent area in the region 160 µm directly upstream pre‐constriction (1000 s^−1^) and 160 µm directly downstream post‐constriction (2000, 3000, or 5000 s^−1^) (Figure 3A, dotted boxes). For all three constricting channels tested, significantly more platelets adhered in the pre‐constriction (low shear) region, than the post‐constriction (high shear) region on both PVC (all *P* < 0.01) and PC (all *P* < 0.05) (Figure 3F,G). When compared to the continuous flow channel at 1000 s^−1^, platelet adhesion at the pre‐constriction 1000 s^−1^ region was not significantly different, however, there was significantly less platelet adhesion in the post‐constriction high shear regions of all three constricting channels on both PVC (all *P* < 0.05) and PC (all *P* < 0.01) (Figure 3F,G). These results are in agreement with the previous section showing significantly decreased platelet adhesion at regions exceeding 1000 s^−1^ (Figure 2D,F). No statistically significant differences were observed between PVC and PC (Figure S3B, Supporting Information).

### 2.4 Platelet Adhesion Increased after Decelerating Flow Regions

**Figure 4:**
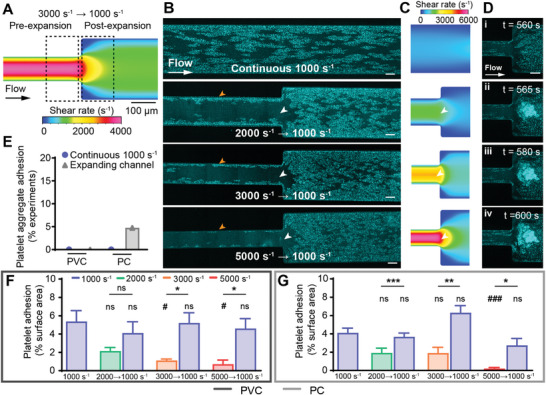
Platelet adhesion increased after decelerating flow regions. A) Schematic of the 3000 to 1000 s^−1^ expanding microfluidic, with dotted boxes representing areas for statistical analysis. B) Representative confocal micrographs of platelet adhesion (cyan) on PVC after 10 min of perfusion, at continuous flow, or decelerating from 2000, 3000, and 5000 s^−1^ to 1000 s^−1^. Scale bar = 50 µm. C) CFD model of the wall shear rate in tested expanding microfluidic channels. D) Representative confocal micrograph time‐course of a 3000 to 1000 s^−1^ PC microfluidic channel showing platelet aggregate adhesion and occlusion. Scale bar = 50 µm. E) Percentage of experiments with platelet aggregate adhesion in continuous and constricting microfluidic channels on PVC (n = 0/19 and n = 0/24 respectively) and PC (n = 0/21 and n = 1/22 respectively). Analysis of total fluorescent surface area indicating platelet adhesion at 10 min on F) PVC and G) PC expanding channels. Error bars are mean ± SEM. *n* = 5 donors who did not show platelet aggregation. Significance comparisons between pre‐ and post‐expansion platelet adhesion for each expanding channel are *P < 0.05, **P < 0.01, ***P < 0.001 by a paired Student's *t*‐test. Significance comparisons to the continuous 1000 s^−1^ channel are #*P* < 0.05, ##*P* < 0.01, ###*P* < 0.001 by an ordinary one‐way ANOVA with Bonferroni's post hoc test.

To further investigate the effect of changing shear gradients on platelet adhesion in our ECMO thrombosis‐on‐a‐chip model, expanding microfluidic channels were used to mimic the downstream region of TCJs, where flow exits the connector (smaller diameter) into tubing (larger diameter), causing flow deceleration and a decrease in the shear rate (Figure 1E,F and **Figure** **4**A). Three expanding‐flow shear gradients of, 2000 to 1000 s^−1^, 3000 to 1000 s^−1^, and 5000 to 1000 s^−1^, were compared to continuous shear at 1000 s^−1^ as per the previous section.

In the high‐shear, pre‐expansion region of all three channels (2000, 3000 and 5000 s^−1^), platelets adhered in a monolayer at the near‐wall (approaching 0 s^−1^), low‐shear region (Figure 4B, orange arrowhead). At the expansion point, there was an area of scarce platelet adhesion (Figure 4B, white arrowheads) as the shear rate transitioned from high to low (Figure 4C, white arrowheads), similar to that observed in constricting conditions (Figure 3B, white arrowheads). Post‐expansion, the 1000 s^−1^ regions adhered platelets in a monolayer of clusters in a similar pattern to the continuous 1000 s^−1^ channel (Figure 4B, Figure S4C, Supporting Information), and 1000 s^−1^ pre‐constriction region constricting channels (Figure 3B).

In the expanding channels, aggregate adhesion (Figure 4D) was not observed in any PVC channels (n = 0/24) and only observed in one PC channel (n = 1/22) (Figure 4E). In one 5000 to 1000 s^−1^ expanding PC channel, a large platelet aggregate adhered to the biomaterial sample at the expansion point (Figure 4Dii). The aggregate rapidly bound additional platelets until it formed a large occlusive clot (Figure 4Div). There was no sign of platelet detachment or embolism over the recorded period (Movie S3, Supporting Information).

Analysis of five independent donor experiments that did not adhere platelet aggregates showed significantly increased platelet adhesion in the post‐expansion (low shear) region compared to the pre‐expansion (high shear) region, except from 2000 to 1000 s^−1^ on PVC (Figure 4F,G). When compared to the continuous flow channel at 1000 s^−1^, the post‐expansion 1000 s^−1^ region had no significant difference in platelet adhesion (Figure 4F,G). Meanwhile, compared to continuous flow at 1000 s1 the 3000 s^−1^ pre‐expansion regions on PVC (*P* < 0.05), and the 5000 s^−1^ pre‐expansion region showed significantly less platelet adhesion on both PVC (*P* < 0.05) and PC (*P* < 0.01) (Figure 4F,G). Again, this is consistent with previous sections showing a statistically significant increase in platelet adhesion at regions below 1000 s^−1^ (Figure 2C,E and Figure 3F,G). No statistically significant differences were recorded between PVC and PC (Figure S3C, Supporting Information).

### 2.5 Stopping Flow Followed by 1000 s^−1^ Increased Platelet Adhesion and Rate of Adhesion on PVC

Clinically, during ECMO the blood pump and flow may be temporarily stopped during treatment for rapid circuit component changeover. To simulate this effect in our model, we paused flow in our microfluidics from 2–4 min (we termed paused flow, 1000 to 0 to 1000 s^−1^). We compared this to continuous flow (as in our prior experiments), as well as having the flow stopped and allowing the blood to diffuse into the channel between 0–2 min before starting flow from 2–10 min (we termed no flow, 0 to 1000 s^−1^). We investigated this in the microfluidic model at 1000 s^−1^ to mimic the shear rate in I.D. = 3/8″ tubing at 4 L min^−1^.

**Figure 5:**
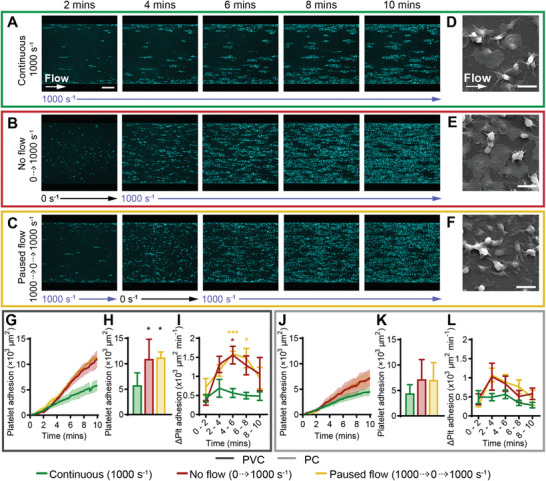
Stopping flow followed by 1000 s^−1^ increased platelet adhesion and rate of adhesion on PVC. Representative confocal micrographs of platelet adhesion (cyan) on PVC over 10 min of flow at A) continuous 1000 s^−1^, B) no flow (0 to 1000 s^−1^) and C) paused flow (1000 to 0 to 1000 s^−1^). Scale bar = 50 µm. Representative scanning electron micrographs of platelet morphology on PVC at the mid‐width of the channel, after 10 min of perfusion at D) continuous 1000 s^−1^, E) no flow and F) paused flow. Scale bar = 5 µm. Total fluorescent surface area indicating platelet adhesion over 10 min on G) PVC and J) PC. Analysis of total platelet adhesion at 10 min on (H) PVC showed statistically significant increase from continuous 1000 s^−1^ compared to no flow and paused flow conditions, K) PC showed no statistically significant differences. Analysis of platelet adhesion rate over 2‐minute periods showed I) significantly increased rate of adhesion for paused flow at *t* = 4 – 8 min compared to continuous 1000 s^−1^ flow on PVC, L) no statistically significant difference on PC. Error bars are mean ± SEM, *n* = 5 donors. Significance comparisons are *P < 0.05, **P < 0.01, ***P < 0.001 by an ordinary one‐way ANOVA (H,K) or two‐way ANOVA (I,L) with Bonferroni's post hoc test. (H,K) Significance presented compared to continuous 1000 s^−1^ flow unless otherwise indicated. (I,K) Red asterisk compared no flow to continuous 1000 s^−1^ flow, yellow asterisk compared paused flow to continuous 1000 s^−1^.

Continuous flow at 1000 s^−1^ adhered platelets in a monolayer at near‐wall regions, and discrete monolayer clusters in the mid‐channel region (**Figure** **5**A). For the no flow experiment (0 to 1000 s^−1^), during no flow (0 s^−1^, t = 0 – 2 min), blood diffused into the saline primed channel and discrete platelets adhered evenly across the channel. When flow was started (1000 s^−1^) at t = 2 min, these platelets remained adhered to the biomaterial, and additional platelets adhered adjacent to existing platelets in clusters until most of the channel was covered in a platelet monolayer at *t* = 10 min (Figure 5B). In the paused flow experiment (1000 to 0 to 1000 s^−1^), at 1000 s^−1^ (between t = 0 – 2 min), platelets adhered to the surface in a continuous monolayer at near‐wall regions and discrete streaks in the mid‐channel. When flow was paused (0 s^−1^, t = 2 – 4 min), residual fluid motion caused deceleration of blood into the channel and discrete platelets adhered across the width of the channel. After flow was restarted (1000 s^−1^, t = 4 – 10 min), platelets adhered adjacent to existing clusters until an almost complete monolayer covered the channel at t = 10 min (Figure 5C). Scanning electron micrographs at the mid‐channel for all conditions showed platelets at all states of platelet activation including round, resting state, early pseudopod formation, spread, and fully spread platelets (Figure 5D–F). Similar observations were recorded for PVC and PC (Figure 5A–; Figure S5A, Supporting Information).

Compared to continuous flow at 1000 s^−1^, no flow and paused flow caused higher platelet adhesion after 10 min (Figure 5G,H,J,K), with statistically significant increases of 1.9 ± 0.7 and 1.9 ± 0.2 ‐fold respectively on PVC (*P* < 0.05) (Figure 5G,H). Additionally, the rate of platelet adhesion between t = 6 – 10 min trended higher for the no flow and paused flow experiments compared to continuous 1000 s^−1^ (Figure 5I,L), with statistically significant increases during the t = 4 – 6 min period for no flow and paused flow, and t = 6 – 8 min period for the paused flow condition on PVC (Figure 5I). After t = 6 min the rate of platelet adhesion started decreasing for both the no flow and paused flow conditions (Figure 5I,L). There were no statistically significant differences in total platelet adhesion on PVC compared to PC (Figure S3D, Supporting Information).

### 2.6 Stopping Flow Followed by 3000 s^−1^ Increased Platelet Adhesion on PVC and PC

**Figure 6:**
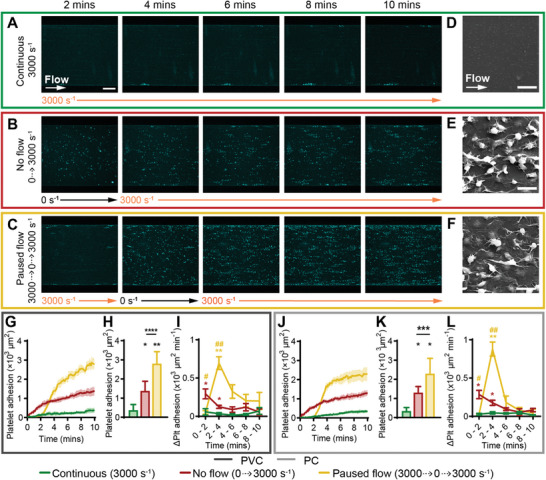
Stopping flow followed by 3000 s^−1^ increased platelet adhesion on PVC and PC. Representative confocal micrographs of platelet adhesion (cyan) on PVC over 10 min of flow at A) continuous 3000 s^−1^, B) no flow (0 to 3000 s^−1^) and C) paused flow (3000 to 0 to 3000 s^−1^). Scale bar = 50 µm. Representative scanning electron micrographs of platelet morphology on PVC at the mid‐width of the channel, after 10 min of perfusion at D) continuous 3000 s^−1^, E) no flow, and F) paused flow. Scale bar = 5 µm. Total fluorescent surface area indicating platelet adhesion over 10 min on G) PVC and J) PC. Analysis of total platelet adhesion at 10 min showed a statistically significant increase from continuous 3000 s^−1^ to 0 to 3000 s^−1^ and 3000 to 0 to 3000 s^−1^ conditions, and between 0 to 3000 s^−1^ to 3000 to 0 to 3000 s^−1^, on both H) PVC and K) PC. Analysis of platelet adhesion rate over 2‐minute periods showed no flow significantly increased the rate of adhesion between t = 0 – 4 min and paused flow significantly increased the rate of adhesion between t = 2 – 4 min on both I) PVC and L) PC. Error bars are mean ± SEM, *n* = 5 donors. Significance comparisons are *P < 0.05, **P < 0.01, ***P < 0.001, ****P < 0.0001 compared to continuous 3000 s^−1^, or #P < 0.05, ##P < 0.01 compared to no flow (0 to 3000 s^−1^) by an ordinary one‐way ANOVA (H,K) or two‐way ANOVA (I,L). (H,K) Significance presented compared to continuous 1000 s^−1^ flow unless otherwise indicated). (I,L) Red asterisk compared no flow to continuous 1000 s^−1^ flow, yellow asterisk compared paused flow to continuous 1000 s^−1^, yellow hash compared paused flow to no flow.

Following the previous section, the effect of stopping ECMO circuits at higher flowrates or in the narrow tubing connector regions was investigated, by starting and stopping the flow at the same intervals, at a shear rate of 3000 s^−1^. After 10 min of continuous flow at 3000 s^−1^, few discrete platelets adhered at the channel wall only (**Figure** **6**A). For the no flow (0 to 3000 s^−1^) condition, during no flow (0 s^−1^, t = 0 − 2 min), platelets adhered evenly across the width of the channel similarly to the 0 to 1000 s^−1^ experiment. When flow started (3000 s^−1^) at t = 2 min, most of the platelets remained adhered and additional platelets adhered in streaks in the direction of flow, resulting in a dispersed monolayer of platelet clusters across the channel at t = 10 min (Figure 6B). At the paused flow (3000 to 0 to 3000 s^−1^) condition, at 3000 s^−1^ between t = 0 – 2 min, few platelets adhered confined to the channel wall only. Paused flow (0 s^−1^, t = 2 – 4 min) caused deceleration of blood in the channel and discrete platelet deposition across the channel. When flow was reinstated (3000 s^−1^) from t = 4 min, platelets adhered adjacent to existing platelets, forming a monolayer of dispersed platelet clusters at t = 10 min (Figure 6C). PVC and PC showed similar observations in platelet adhesion (Figure 6A–C; Figure S5, Supporting Information). Scanning electron micrographs at the mid‐channel region of the continuous flow device (≈3000 s^−1^) showed no platelet adhesion (Figure 6D). At the no flow and paused flow conditions, platelets were observed at all stages of platelet activation in the mid‐channel regions. Platelets in early pseudopod formation appeared to form a long pseudopod trailing behind the platelet in the direction of flow (Figure 6E,F).

Compared to continuous flow at 3000 s^−1^, the no flow and paused flow conditions increased platelet adhesion after 10 minutes significantly by 3.7 ± 1.3 and 7.5 ± 1.7 ‐fold respectively on PVC (Figure 6G,H) and 3.8 ± 0.9 and 6.7 ± 2.3 ‐fold respectively on PC (Figure 6J,K). The rate of platelet adhesion when the pump was turned off in the paused flow condition (PVC: 696.6 ± 83.90, PC: 879.3 ± 203.88 ×103 µm min^−1^), trended higher compared to when the pump was off in the no flow condition (PVC: 294.8 ± 64.4, PC: 286.1 ± 55.48 µm min^−1^) on both materials (Figure 6I,L), suggesting that paused flow adheres platelets at an increased rate compared to stagnant flow. No significant difference between total platelet adhesion was recorded between PVC and PC for the tested conditions after 10 min (Figure S3E, Supporting Information).

### 2.7 Platelet Activation Marker P‐Selectin was Increased on PVC Compared to PC

**Figure 7:**
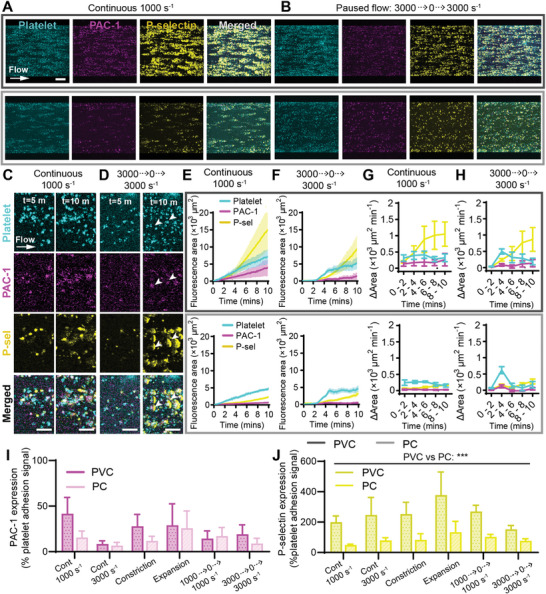
Platelet activation marker P‐selectin expression was increased on PVC compared to PC. Representative confocal micrographs of total platelet adhesion (cyan) and platelet activation markers PAC‐1 (magenta) and P‐selectin (yellow) expression on PVC (top row) and PC (bottom row) after 10 min at flow conditions of A) continuous 1000 s^−1^ and B) 3000 to 0 to 3000 s^−1^. Scale bar = 50 µm. Representative confocal micrographs showing platelet, PAC‐1, and P‐selectin co‐localization or spreading at t = 5 min compared to t = 10 min at flow conditions of C) continuous 1000 s^−1^ and D) 3000 to 0 to 3000 s^−1^. Scale bar = 5 µm. Total fluorescent surface area indicating platelet adhesion, PAC‐1, and P‐selectin expression over 10 min on PVC (top row) and PC (bottom row) at flow conditions of E) continuous 1000 s^−1^ and F) 3000 to 0 to 3000 s^−1^. Platelet adhesion, PAC‐1, and P‐selectin expression rate over 2‐minute periods at flow conditions of G) continuous 1000 s^−1^ and H) 3000 to 0 to 3000 s^−1^. I) PAC‐1 and J) P‐selectin expression as a percentage of platelet core signal at t = 10 min, analyzed between PVC compared to PC, and between all tested flow conditions, showed that PAC‐1 and P‐selectin expression was independent of flow condition, but PVC caused more P‐selectin expression than PC. Error bars are mean ± SEM, *n* = 5 donors. Significance comparisons in I,J by an ordinary two‐way ANOVA with Bonferroni's post hoc test. Significance comparisons are *P < 0.05, **P < 0.01, ***P < 0.001.

Reducing platelet adhesion to ECMO surfaces is of interest as platelet adhesion can trigger platelet activation and the release of agonists including adenosine diphosphate (ADP) and thromboxane A2 which induces platelet aggregation and activation of the coagulation cascade (Figure S6, Supporting Information).^[^ ^4^ ^]^ The platelet activation state at six flow conditions on PVC and PC (**Figure** **7**; Figure S7, Supporting Information) was monitored by measuring PAC‐1, which specifically binds to activated glycoprotein (GP) IIb/IIIa on platelets, and P‐selectin, a platelet adhesion membrane protein released by platelet alpha granules upon activation which allows binding to leukocytes (Figure S6, Supporting Information).^[^ ^22^ ^]^ To investigate platelet activation status, we used whole blood heparinised at 5 U mL^−1^, which although is more time sensitive for in vitro experimentation, is more clinically relevant as patients receive heparin when on ECMO.^[^ ^2^, ^9^ ^]^ Additionally, heparin may be more sensitive to differentiating platelet activation states on different biomaterials than citrated whole blood, though our results showed no significant difference in total platelet adhesion (Figure S2, Supporting Information) nor pattern (Figures 2, 3, 4, 5, 6; Figure S7, Supporting Information), compared to citrated blood in this model.

Platelet adhesion pattern (Figure 7A,B; Figure S7, Supporting Information) and total platelet area (Figure 7E,F; Figure S8A, Supporting Information) over 10 min correlated to earlier experiments (Figures 2, 3, 4, 5, 6). For all tested conditions, at initial platelet adhesion, PAC‐1 and P‐selectin expression were co‐localized with the core of the platelet (Figure 7C,D). Over the 10‐minute experiment on both PVC and PC, PAC‐1 remained co‐localized to the central core of the platelet (Figure 7A–D; Figure S7, Supporting Information). Although total PAC‐1 fluorescence area increased over the course of the experiment, it remained consistently lower than the total platelet signal at all flow conditions (Figure 7E,F; Figure S8A, Supporting Information). The stable, positive rate of adhesion indicated by the change in fluorescence area over time intervals (Figure 7G,H; Figure S8B, Supporting Information) was observed to be new PAC‐1 positive platelets adhering to the biomaterial over time, as opposed to existing adhered platelets increasing their expression of PAC‐1 (Movie S4, Supporting Information). After 10 min of flow, 8.3 – 41.5% of platelets on PVC and 6.7 – 25.7% of platelets on PC were positive for PAC‐1 (Figure 7I). There was no significant difference between PAC‐1 expression as a percentage of total platelet signal between PVC and PC, nor between flow conditions, suggesting that PAC‐1 expression was independent of biomaterial and flow at the tested conditions (Figure 7I).

Meanwhile, there were more P‐selectin than PAC‐1 positive platelets at all flow conditions on both PVC and PC (Figure 7E,F, Figure S8A, Supporting Information). P‐selectin was initially co‐localized to the platelet core (Figure 7C,D, t = 5 min), but spread around the platelet over time (Figure 7C,D, t = 10 min). Additionally, in the paused flow 3000 to 0 to 3000 s^−1^ condition at the high‐shear mid‐channel region, some P‐selectin shifted downstream toward the leading edge of the platelet over time, presenting as long streak perpendicular to the direction of flow on both PVC and PC (Figure 7D, white arrowheads, Movie S4, Supporting Information). As P‐selectin spread around the platelet core, this was seen as a sudden increase in total P‐selectin signal ≈4 min after the experiment start, and P‐selectin signal exceeded that of the total platelet signal over time on PVC (Figure 7E,F; Figure S8A, Supporting Information). The high rate of P‐selectin expression appeared to increase from the t = 4 – 6 min period onwards, on both PVC and PC (Figure 7G,H; Figure S8B, Supporting Information). After 10 min, there was no significant difference in P‐selectin expression between flow conditions, however, total P‐selectin fluorescence area was significantly increased on PVC (152.4.3 – 377.2% of the platelet core area) compared to PC (48.7 – 133.5% of platelet core area, P < 0.001) (Figure 7J).

## 3 Discussion

Our ECMO thrombosis‐on‐a‐chip model showed that shear regimes correlating to ECMO circuit flow conditions affect acute platelet adhesion to the circuit, while the biomaterial affects platelet activation. Human whole blood was flowed over PVC and PC biomaterial samples in a microfluidic channel, at shear rates of 500 – 5000 s^−1^ to mimic ECMO tubing and connectors at 2 – 6 L min^−1^, as verified by CFD modeling. On both PVC and PC, low shear rates (500 and 1000 s^−1^) significantly increased platelet adhesion compared to high shear rates (2000, 3000, and 5000 s^−1^). Similarly, increased platelet adhesion was recorded in the lower‐shear (1000 s^−1^) regions of constricting and expanding microfluidic channels mimicking ECMO TCJs, and platelet aggregate stabilization, growth and embolism were visualized in these channels. Furthermore, stopping and restarting blood flow in the channel increased platelet adhesion compared to continuous flow. Finally, expression of the platelet activation marker P‐selectin was increased on PVC compared to PC. The results suggested that operationally, low shear and flow stasis should be reduced during ECMO where possible to decrease platelet adhesion, and demonstrated the ECMO thrombosis‐on‐a‐chip model's ability to delineate the role of flow and biomaterial properties on platelet adhesion and activation in the initial stages of clinically relevant biomaterial thrombosis.

To maintain normal hemostatic function in ECMO patients, ECMO biomaterial and flow conditions should be designed to reduce both platelet activation which would trigger fibrin polymerization and thus clot growth,^[^ ^22^, ^23^, ^24^ ^]^ while reducing platelet dysfunction that would cause bleeding.^[^ ^6^, ^25^ ^]^ Currently, research of ECMO thrombosis mechanisms and exploring methods for improving patient treatment are limited as traditional *ex vivo* and in vitro models do not allow high‐throughput testing at clinically relevant conditions. Mock circulation loops are commonly used,^[^ ^26^ ^]^ however these models require high volumes of blood (> 500 mL per experiment) meaning throughput is low, and often requires large animal blood which is physiologically different to humans.^[^ ^26^, ^27^ ^]^ As our model requires on average 300 µL of blood per experiment, this allows rapid screening of various blood flow and biomaterial interactions using human blood donations. Other dynamic hemocompatibility testing platforms such as Chandler loops, TEG/ ROTEM and rheometers do not allow real‐time monitoring of dynamic blood states and interactions, are challenging to incorporate customizable medical device relevant flow conditions and biomaterials, and require larger blood volumes than microfluidics, limiting their applications and output.^[^ ^15^, ^28^ ^]^ Therefore, by combining CFD with microfluidics, the ECMO thrombosis‐on‐a‐chip model provides a tailorable biomaterial and flow, higher‐throughput model of human whole blood thrombosis under ECMO conditions that mimics clinical scenarios.

To our knowledge, this is the first study to investigate human whole blood platelet adhesion and activation on polymeric biomaterials at selected ECMO‐relevant shear rates and flow conditions. Our system showed increased platelet adhesion below 1000 s^−1^ with maximum platelet adhesion at 500 s^−1^, as well as increased platelet adhesion at decelerating velocity gradients corresponding to decreased shear rates. These results correlate with in silico simulations that platelet margination toward surfaces increases up to around 500 s^−1^, but negligible changes beyond 1000 s^−1^.^[^ ^29^, ^30^ ^]^ Furthermore, studies have demonstrated that platelet adhesion on fibrinogen coated surfaces was maximum around 500 s^−1^,^[^ ^31^, ^32^ ^]^ which correlates with our results, suggesting that fibrinogen is mediating platelet adhesion to biomaterials in our model. This is in agreement with well‐established literature that initial platelet adhesion to biomaterials is mediated by surface adsorbed fibrinogen under similar flow conditions.^[^ ^3^, ^33^ ^]^ Studies on von Willebrand's factor (vWF) coated surfaces showed large platelet aggregate formation at shear rates above 3000 s^−1^,^[^ ^16^, ^34^, ^35^, ^36^ ^]^ thus our result of reduced platelet adhesion at 3000 and 5000 s^−1^ implies that after 10 min, PVC and PC had not adsorbed vWF which would cause large platelet aggregate formation in the channel.

We recorded no significant difference in platelet adhesion between PVC and PC, however, interestingly we observed increased P‐selectin expression on PVC compared to PC, but not PAC‐1. There is limited literature on platelet receptor activation over time on biomaterials under flow, therefore, our ability to visualize changes in expression of platelet activation markers over time in the ECMO thrombosis‐on‐a‐chip model allows us to explore the activation status of platelets for the first time. The low level of PAC‐1 shows the majority of the platelet integrin GPIIb/IIIa is not in its active form on the platelets bound to either PVC or PC, and indicates that these platelets are not fully activated to aggregate, and are in a biomechanical intermediate activation state.^[^ ^37^ ^]^ The increasing levels of P‐selectin over the course of the experiment indicates platelet degranulation occurs in the first 10 min of adhesion to the materials.^[^ ^3^, ^23^ ^]^ Differential platelet P‐selectin expression has previously been observed in bulk clots, with P‐selectin positive platelets tightly adhered in the core region of the thrombus and platelets at the outer edges of the thrombus that are P‐selectin negative tend to embolize.^[^ ^24^ ^]^ To our knowledge, this is the first observation of P‐selectin expression at the leading edge of platelets bound to biomaterials under high shear conditions relevant to ECMO circuits (or any single platelet expression of P‐selectin under flow by microscopy). High P‐selectin at the leading edge indicates preferential release of alpha granules at the leading edge, the contents of which could promote further platelet adhesion, activation and coagulation as they contain vWF, platelet integrins, fibrinogen and coagulation factors.^[^ ^38^ ^]^ Together, the ability of our model to monitor multiple platelet activation markers and visualize their spatial distribution, allows further exploration of mechanisms of platelet activation on biomaterials, by integrating P‐selectin, PAC‐1, or other receptor blockers to identify the receptors required for biomaterial platelet adhesion and activation.

With regards to the higher P‐selectin on platelets on PVC compared to PC, both are hydrophobic polymers with similar wettabilities, and though PVC and PC were both essentially smooth, PVC was ≈4 nm rougher than PC. Previous studies of human platelet adhesion showed no difference in platelet adhesion on surfaces with roughness in the range of 1 – 95 nm,^[^ ^39^, ^40^, ^41^, ^42^ ^]^ and increased platelet adhesion only when surfaces were rougher by or contained topographical features of 120 – 400 nm.^[^ ^43^, ^44^, ^45^ ^]^ Meanwhile, on some materials, platelet activation increased with increasing roughness (1 – 400 nm), however, this trend is not always seen, likely due to the difference in other material properties.^[^ ^41^, ^46^ ^]^ Therefore, further systematic investigation is required to isolate the role of material properties such as topography, chemistry, charge or stiffness, on platelet activation under flow on PVC compared to PC. Furthermore, although we observed no significant differences between platelet activation states at our selected flow conditions, a wider range of tested flow conditions would reveal differences in platelet activation states.^[^ ^25^, ^47^, ^48^ ^]^ These results highlight the need for further investigation of platelet activation states, mechanobiology and mechanosensing pathways^[^ ^49^ ^]^ in relation to biomaterial platelet adhesion, aggregation, embolism and occlusion under different biomaterial and flow conditions to understand how to prevent the initiation and activation of thrombosis.

The results obtained here from the ECMO thrombosis‐on‐a‐chip model replicated known clinical observations,^[^ ^50^, ^51^ ^]^ supporting its use for informing future ECMO operation, design, and material selection. We recorded complete biomaterial surface coverage by platelets at 500 s^−1^, which aligns with the recommended minimum clinical ECMO flowrate of 2 – 2.5 L min^−1^ below which increased clotting complications are recorded under current biomaterial and anticoagulant standards.^[^ ^2^ ^]^ Therefore, our model may be used to determine whether this threshold changes with future antithrombotic biomaterials, novel anticoagulants, or patient specific anticoagulation or hemostatic conditions, and could guide ECMO operation. Additionally, blood flow stasis and circuit pausing are already minimised during circuit changeovers, and stasis and deceleration have been well‐established to increase clotting in clinical, *ex vivo* and in vitro studies.^[^ ^50^, ^51^ ^]^ This aligns with a proposed and tested change to ECMO connector design that removes connector barbs to reduce flow acceleration and deceleration zones where thrombosis is observed.^[^ ^52^ ^]^ In terms of ECMO material selection, the result that PVC caused increased platelet activation compared to PC suggests PVC ECMO components that do not require flexibility can be replaced with PC to reduce platelet activation. Such changes have been seen without impacting ECMO functionality in the Maquet HLS 7.0 pump which has a longer PC inflow (> 10 cm of PC inflow, compared to ≈2 cm in the previous Maquet Quadrox PLS),^[^ ^53^ ^]^ which also additionally improved ECMO transport and portability. These types of changes could be informed by our model, but ultimately need to be designed in consultation with engineers, clinicians, and patient carers to ensure ECMO functionality is not affected.

Beyond ECMO design changes, the ECMO thrombosis‐on‐a‐chip model's ability to replicate clinical scenarios also provides the opportunity to monitor underexplored clinical issues such as embolism and occlusion^[^ ^5^, ^19^ ^]^ which currently cannot be studied in real‐time, thus little is known about their source and manifestation. We recorded platelet aggregates causing embolism and occlusive thrombi at shear gradients in real‐time, which means this model could be used to visualize and analyze mechanisms of embolism including shear thresholds which cause embolism as opposed to occlusive thrombosis, time to embolism, size of emboli, and whether certain biomaterials are more prone to causing embolism than others. However, we could not verify whether these aggregates were formed during blood collection or under flow in the channel, and establishing a method to reproducibly form controlled platelet aggregates, or a model that spontaneously forms embolism, would be required. Furthermore, compared to our results showing increased P‐selectin on platelets in our ECMO thrombosis‐on‐a‐chip model, little is known about the expression of P‐selectin on platelets bound to ECMO circuits. Only studies of circulating platelet activation states during ECMO have been carried out, showing conflicting data with regards to P‐selectin expression.^[^ ^54^, ^55^ ^]^ As these studies evaluated circulating blood samples collected from the ECMO circuit or the patient via flow cytometry, it does not provide information on whether the ECMO biomaterials or flow conditions have caused the paradoxical effects of platelet activation, thrombosis or high shear induced platelet dysfunction.^[^ ^25^, ^56^ ^]^ Therefore, the ECMO thrombosis‐on‐a‐chip model provides the opportunity to identify the specific isolated biomaterial or flow conditions that promote activation of specific platelet receptors or dysfunction over time and compare this to clinical samples.

Overall, we have shown that the ECMO thrombosis‐on‐a‐chip model is a powerful tool for highly customizable testing of flow and biomaterial mediated thrombosis, at a higher‐throughput with lower blood volume than was previously possible. The ability to monitor multiple blood components in real‐time can advance the field's knowledge of the biological processes of biomaterial thrombosis under various conditions. For medical device engineers, prothrombotic flow and shear thresholds can be identified with this system and used to inform future medical device design, while biomaterial researchers can test novel biomaterials and coatings under operationally relevant flow conditions. Additionally, the model can be used to assist clinical knowledge by analyzing how total platelet adhesion and activation differs between novel or different anticoagulant treatments, drug interactions, patient comorbidities, and blood damage from ECMO. The model could also be used to investigate ECMO thrombosis in patients who cannot receive anticoagulants, where we expect rapid fibrin polymerization and channel occlusion even at high shear rates, as observed in our model with recalcified blood conditions. Furthermore, this model could inform the propensity of thrombosis in new microfluidic based medical devices, such as recently developed microfluidic oxygenators for extracorporeal circuits, which showed improved gas transfer efficiency^[^ ^57^, ^58^ ^]^ and eliminated the need blood damaging centrifugal pumps.^[^ ^59^ ^]^ Ultimately the ECMO thrombosis‐on‐a‐chip model could guide safety improvements in the next‐generation of blood‐contacting biomaterials and medical devices by reducing clinical complications from thrombosis and bleeding.

## 4 Limitations

While this investigation focused on acute blood‐biomaterial responses to characterize and understand thrombus initiation, whole blood is only stable *ex vivo* for a few hours so answering the longer‐term effects of blood flow on the biomaterial adsorbed protein layer and thrombus formation over the course of days would still require in vivo large animal models. Additionally, these experiments do not take into account that patients on ECMO have impaired hemostatic and inflammatory function, and ECMO thrombosis and embolism can develop over multiple days during patient treatment, and the prolonged blood exposure to pathological shear in circuits (pathological ECMO range of 0 – >10000 s^−1^,^[^ ^60^ ^]^ compared to physiological healthy human range of 20–2000 s^−1[^ ^61^ ^]^) causes disorders including acquired vWF syndrome, platelet activation and dysfunction, thrombocytopenia, and hemolysis.^[^ ^6^, ^7^, ^55^, ^62^ ^]^ Conducting experiments with patient blood samples would be necessary to appropriately investigate these effects. Additionally, some ECMO relevant flow features such turbulence, and the size of flow gaps relative to the blood cell and protein sizes, cannot be replicated at the micro‐scale, thus, studies with full scale models are still required to study these effects. In addition, the system presented here used unplasticized PVC, whereas clinical‐grade ECMO PVC tubing contains plasticizer such as noDOP (triethylhexyl trimellitate (TEHTM)) for flexibility. TEHTM plasticized PVC is hydrophobic,^[^ ^63^ ^]^ similar to the unplasticized PVC used here, however, there is not enough information regarding noDOP's composition and concentration available to determine its effect on other material surface properties. Incorporation of clinically used PVC tubing into microfluidic chips for these real‐time imaging studies is challenging due to its curvature and thick walls, and would require extensive engineering and optimisation beyond the scope of this study.

## 5 Conclusion

We have developed a microfluidic ECMO thrombosis‐on‐a‐chip model integrating clinically relevant flow and customizable biomaterial combinations in a low blood volume, high‐throughput model system that can be used to evaluate platelet adhesion and activation in real‐time. Using this model, we found increased platelet adhesion at low shear < 1000 s^−1^ and from stopping flow, mimicking clinically observed thrombus formation. We additionally observed increased P‐selectin expression on platelets bound to PVC compared to PC over time, and showed platelet activation occurs in the first 10 minutes of platelet adhesion under flow. These results could inform future ECMO design and clinical operation to reduce platelet adhesion and activation that initiates thrombotic complications, as well as providing a model system for future investigation of the biological mechanisms underlying biomaterial thrombosis and evaluation of novel blood‐contacting biomaterials under clinically relevant flow conditions.

## 6 Experimental Section

### Identification of ECMO Thrombosis

Discarded patient ECMO circuits (*n* = 2 circuits) were obtained from Royal Prince Alfred Hospital, NSW, Australia in accordance with the Research Ethics & Governance Office, Royal Prince Alfred Hospital (X23‐0075 & 2023/STE00624: Microfluidics for ECMO and CPB Haemocompatability (MECH 2 Study)). Circuits were immediately flushed with saline post‐decannulation and photographed for thrombi.

### Computational Fluid Dynamics

Computational fluid dynamic modeling was conducted with ANSYS 2021 R1 (FLUENT) (ANSYS, PA, USA), using Microsoft Windows 10 Enterprise Version 10.0.19045 Build 19 045 with an 11^th^ Gen Intel(R) Core (TM) i9‐11950H @ 2.60 GHz, 2611 MHz, 8 Core(s), 16 Logical Processor(s) and 32GB RAM. Standard ECMO tubing (I.D. 3/8″) was modelled as a cylindrical tube 30 cm long (1.5 × fluid entrance length). A barbed tubing connector (O.D. 3/8″) geometry was measured and modelled from an ECMO circuit connector ref. number 05225 D673 – 3/8″ – 3/8″ (Sorin Group, MO, Italy), with cylindrical tubing (I.D. 3/8″) attached at inlet and outlets. The tubing and TCJ were meshed into simplified 2D axisymmetric segments of 200 × 200 µm quadrilateral elements with an inflation boundary layer of first layer thickness = 50 µm and growth rate = 1.2. Flow in the tubing and TCJ were modelled with the k‐omega turbulence model,^[^ ^64^ ^]^ and flow in the microfluidic was modelled as laminar. The density and viscosity of whole blood were approximated as 1060 kg m^−3^ and 0.00345 Pa s respectively, and assumed to be a Newtonian fluid.^[^ ^65^ ^]^ Results obtained by CFD were verified against theoretical calculations where available, mesh independent and calculated to a convergence continuity residual of 10^−6^.

### Wall Shear Rate Calculations

The wall shear rate ($\dot{\gamma }$, in s^−1^) in microfluidic channels was theoretically calculated using the derived Hagen‐Poiseuille formula for wall shear rate in rectangular cross‐sections:^[^ ^20^ ^]^

(1) $$\dot{\gamma }=\mu \cdot \frac{Q\;P\;\lambda }{8A^{2}}$$

where μ is the fluid dynamic viscosity (kg m^−1^ s^−1^), *Q* is the volumetric flow rate (m^3^ s^−1^), *A* is the cross‐sectional area (m^2^), *D_h_* is the hydraulic diameter (m), *P* is the wetted perimeter (m), and λ is the dimensionless shape factor, calculated as below for a rectangular cross‐section:

(2) $$\lambda =\frac{24}{{\left[\left(1-0.351\frac{b}{a}\right)\left(1+\frac{b}{a}\right)\right]}^{2}}$$

where *a* and *b* are the channel dimensions (m).

### Material Wettability Characterization

Material surface wettability was measured via contact angle goniometry (Theta Flex, Attension, Biolin Scientific, Stockholm, Sweden), and analyzed with its inbuilt software OneAttension. The contact angle of a 5 µL Milli‐Q water droplet was recorded at 3 locations on 3 samples per material (n = 9).

### Surface Topography Characterization

Surface topography and roughness were characterized by atomic force microscopy (Bruker Dimension Icon Atomic Force Microscope, MA, USA), and analyzed with NanoScope Analysis 1.9 (Bruker, MA, USA). The root mean square average of height deviation (Rq) of a 10 × 10 µm scan area was recorded at 3 locations on 3 samples per material (*n* = 9).

### Human Whole Blood Collection

Fresh human whole blood was obtained from donors (male or female, 18–60 years old) with informed consent, in accordance with guidelines of The University of Sydney Office for Research Integrity, Human Ethics Committee (Project 2014/244) and the Declaration of Helsinki. Donors were self‐reported as healthy and not taken anticoagulant, anti‐inflammatory or antibiotic medication 72 h prior to donation. Blood was drawn via standard venepuncture into polypropylene syringes (Terumo, Tokyo, Japan) at 3.8% w/v trisodium citrate (Sigma‐Aldrich, MO, USA) (Figures 2, 3, 4, 5, 6; Figures S4 and S5, Supporting Information) or 5 U mL^−1^ heparin sodium (Pfizer, NY, USA) (Figure 7; Figure S7, Supporting Information), both in 10% w/v saline in whole blood and stored on a 37 °C heat block until use.

### Human Whole Blood Fluorescent Labeling

Citrated whole blood platelets were labelled with 0.5 µg mL^−1^ DiOC6(3) (D273, Invitrogen; Thermo Fisher Scientific, MA, USA) from a solution of 1 µg mL^−1^ in DMSO (Sigma Aldrich, MO, USA) (final dilution of 0.05% v/v per mL of blood). For studies of platelet activation states, heparinised whole blood was used, and platelets were labelled as above, PAC‐1 with 6.66 µL mL^−1^ Alexa Fluor anti‐human CD41/CD61 antibody from stock ^[^ ^66^ ^]^ (362 806, Clone: PAC‐1, Lot: B377638, concentration: 150 µg mL^−1^, formulation: phosphate‐buffered solution containing 0.09% sodium azide and bovine serum albumin, BioLegend, CA, USA) and P‐selectin with 7.5 µL mL^−1^ Brilliant Violet 421 anti‐human CD62P (P‐selectin) antibody from stock ^[^ ^66^ ^]^ (304 926, Clone: AK4, Lot: B369274, concentration 100 µg mL^−1^, formulation: phosphate‐buffered solution containing 0.09% sodium azide and bovine serum albumin, BioLegend, CA, USA). For initial screening studies with recalcified citrated blood, 62.5 µL of recalcification buffer (constituting 100 mm CaCl_2_ and 75 mm MgCl_2_, final concentration in blood of 6.25 mm and 4.69 mm respectively, both from Sigma Aldrich, MO, USA) was added per mL of citrated whole blood, gently inverted three times, and incubated at 37 °C for 5 min prior to experimentation. Citrated and heparinised whole blood were used within 3 h and 1.5 h of the blood draw respectively. Fibrin formation was monitored by adding 15 µg mL^−1^ AF647 conjugated fibrinogen from human plasma (F35200, Invitrogen; Thermo Fisher Scientific, MA, USA), in a solution of 1.5 mg mL^−1^ prepared by reconstituting the stock in 0.1 m sodium bicarbonate) to whole blood (final dilution of 1% v/v per mL of blood). All dyes and antibodies were added to whole blood immediately after the blood draw, were gently inverted three times, and incubated at 37 °C for 30 min prior to experimentation. Red blood cell, monocyte, and neutrophil adhesion were monitored by differential interference contrast (DIC) microscopy after flushing the microfluidic channel immediately after the experiment with 0.9% sodium chloride saline (Baxter, IL, USA).

### Microfluidic Device Fabrication

Silicon microfluidic wafers were fabricated by standard photolithography at the Research and Prototype Foundry, The University of Sydney. Standard soft lithography methods were used to fabricate polydimethylsiloxane (PDMS) microfluidic devices, by mixing ten parts Sylgard© 184 silicone elastomer base with one‐part curing agent (Dow Corning Chemical Company, MI, USA), vacuum degassed, then cured at 60 °C for 6 h. Unplasticized polyvinyl chloride (PVC) (CV31‐FM‐000100 0.2 mm, Goodfellow, Cambridge, UK) and polycarbonate (PC) (CT30‐FM‐000110 0.175 mm, Goodfellow, Cambridge UK) sheets were cleaned by ultrasonication in 3 washes of Milli‐Q water for 20 s each. PDMS device inlet and outlet ports were created using 1 mm biopsy punch (Miltex, NJ, USA). The PDMS device was then adhered to the PVC or PC sheet. A polypropylene syringe barrel (Terumo, Tokyo, Japan) was adhered with PDMS and cured onto the microfluidic device inlet, to form a fluid reservoir (Figure 1G).

### Microfluidic Flow Assay

Blood flow was withdrawn by a syringe pump (Legato111, KD Scientific) pulling a 10 mL polypropylene syringe (Terumo, Tokyo, Japan) connected to the microfluidic device outlet via tubing (Tygon ND‐100‐65, I.D. 1/32′, Saint‐Gobain Life Sciences, Courbevoie, France) and a 19 gauge 1.0″ I.D. 0.27″ 90° stainless steel bent needle (Jensen Global, CA, USA) (Figure 1G). The microfluidic channel was primed by drawing degassed 0.9% sodium chloride (Baxter, IL, USA) at 50 µL min^−1^ for 1 min. Priming flow was stopped for 1 min, excess saline removed, then labelled whole blood was added to the microfluidic device fluid reservoir and the syringe pump withdrawal promptly started at the target flow rate. Experiments were excluded where microfluidic chip failure occurred, or if the blood sample was coagulated prior to experimentation.

### Fluorescence Microscopy

Microscopy of blood adhesion on microfluidic materials was conducted on the LSM800 Airyscan Axio Observer.Z1/7 (Zeiss, Oberkochen, Germany). Figures 2, 3, 4, 5, 6 and Figures S1,S4, and S5 (Supporting Information) were acquired using a 25×/0.8 Imm Korr DIC M27 objective, laser wavelength 488 (DiO), 640 (AF647) and 405 (P‐selectin) nm and a gain of 639 (DiO), 648 (AF647) and 650 (P‐selectin) V. Figure 7 and Figures S6 and S7 (Supporting Information) were acquired using a 40×/1.3 Oil DIC (UV) VIS‐IR M27 objective, laser wavelength 488 (DiO), 640 (PAC‐1) and 405 (P‐selectin) nm and gain of 700 (DiO), 650 (PAC‐1) and 600 (P‐selectin) V. The closed microscope chamber and imaging stage were maintained at 37 °C.

### Scanning Electron Microscopy

Microfluidic devices were fixed in 2.5% glutaraldehyde in 0.1 m phosphate buffer (PB) for 30 min (Sigma Aldrich, MO, USA), 1% osmium tetroxide in 0.1 m PB (Electron Microscopy Sciences, PA, USA) for 1 h, dehydrated in ascending grades of ethanol, dried with hexamethyldisilane (Sigma Aldrich, MO, USA), and dried in a desiccator overnight. The samples were mounted and sputter coated with 5 µm of gold and imaged on the Phenom XL Desktop SEM (ThermoFisher Scientific, MA, USA).

### Image Analysis

ImageJ was used to analyze total platelet, PAC‐1, and P‐selectin area, by applying the Analyze Particles command, where signal exceeding a fixed threshold and exceeding an area of 1 µm^2^, was included as positive signal. Platelet, PAC‐1 and P‐selectin rates of adhesion and expression were calculated by fitting a linear regression between two‐minute intervals, using the inbuilt polyfit function on MATLAB R2023a (MathWorks, MA, USA), and obtaining the regression coefficient.

### Statistical Analysis

Microfluidic experiment results were presented as mean ± standard error of the mean of five independent donors (*n* = 5). Data was analyzed using GraphPad Prism (version 9) (GraphPad, CA, USA), with Student's t‐test, ordinary one‐way or two‐way analysis of variance (ANOVA) with Bonferroni's post hoc multiple comparisons test, where *P* < 0.05 was considered statistically significant.

## Conflict of Interest

The authors declare no conflict of interest.

## Supporting information

Supporting Information

Supplemental Movie 1

Supplemental Movie 2

Supplemental Movie 3

Supplemental Movie 4

## Data Availability

The data that support the findings of this study are available from the corresponding author upon reasonable request.
