# Supplementary material for: Platelet Adhesion and Activation in an ECMO Thrombosis‐on‐a‐Chip Model
Source: Adv Sci (Weinh). 2024 May 17;11(30):2401524. doi: 10.1002/advs.202401524 (PMC11321669; doi:10.1002/advs.202401524)
Supplement: Supplementary file 1 — Supporting Information [file ADVS-11-2401524-s002.pdf]

ADVANCED SCIENCE Open Access

Supporting Information

for *Adv. Sci.*, DOI 10.1002/advs.202401524

Platelet Adhesion and Activation in an ECMO Thrombosis-on-a-Chip Model

*Tiffany Goh, Lingzi Gao, Jasneil Singh, Richard Totaro, Ruaidhri Carey, Kevin Yang, Bruce Cartwright, Mark Dennis, Lining Arnold Ju and Anna Waterhouse**

Supporting Information

Platelet adhesion and activation in an ECMO thrombosis-on-a-chip model

Tiffany Goh[1,2,3,4], Lingzi Gao[1,2,3,4], Jasneil Singh[1,2,3,4], Richard Totaro[5,6], Ruaidhri Carey[6], *Kevin Yang[6], Bruce Cartwright[5,7], Mark Dennis[5,8] Lining Arnold Ju[2,3,4,9], Anna Waterhouse[1,3,4]*

[1]School of Medical Sciences, Faculty of Medicine and Health, The University of Sydney, NSW 2006, Australia

[2]Heart Research Institute, Newtown, NSW 2042, Australia

[3]Charles Perkins Centre, The University of Sydney, NSW 2006, Australia

[4]The University of Sydney Nano Institute, The University of Sydney, NSW 2006, Australia

[5]Faculty of Medicine and Health, University of Sydney, NSW 2006 Australia

[6]Intensive Care Department, Royal Prince Alfred Hospital, Missenden Road, Camperdown, Sydney, NSW 2050, Australia.

[7]Anaesthetics Department, Royal Prince Alfred Hospital, Camperdown, Sydney, NSW 2050, Australia

[8]Cardiology Department, Royal Prince Alfred Hospital, Missenden Road, Camperdown, Sydney, NSW 2050, Australia.

[9]School of Biomedical Engineering, Faculty of Engineering, The University of Sydney, Darlington, NSW 2008, Australia

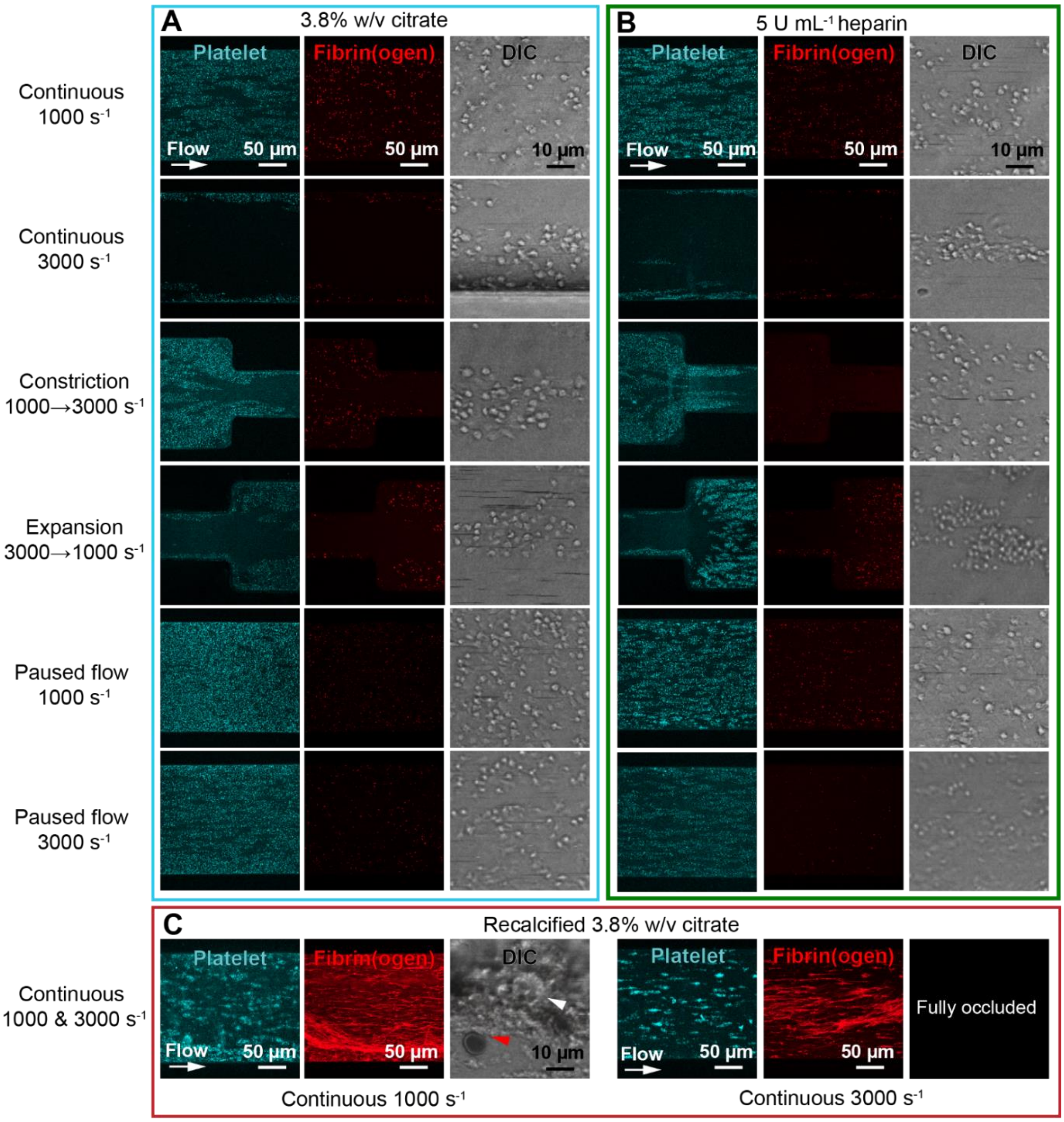

Figure S1. Representative confocal and differential interference contrast (DIC) micrographs of platelet adhesion (DiOC6, cyan), fibrin formation (AF647, red), and monitoring for red blood cell, neutrophil or monocyte adhesion (DIC), at 6 screened flow conditions on PVC at t = 10 min, using **(A)** 3.8% w/v citrated blood, **(B)** 5 U mL^{-1} heparinised blood and **(C)** recalcified 3.8% w/v citrated blood. Only the recalcified citrated blood condition showed polymerised fibrin formation, red blood cell adhesion (red arrowhead) and neutrophil adhesion (white arrowhead). Scale bar = 50 μm (platelets, fibrin/ogen) or 10 μm (DIC).

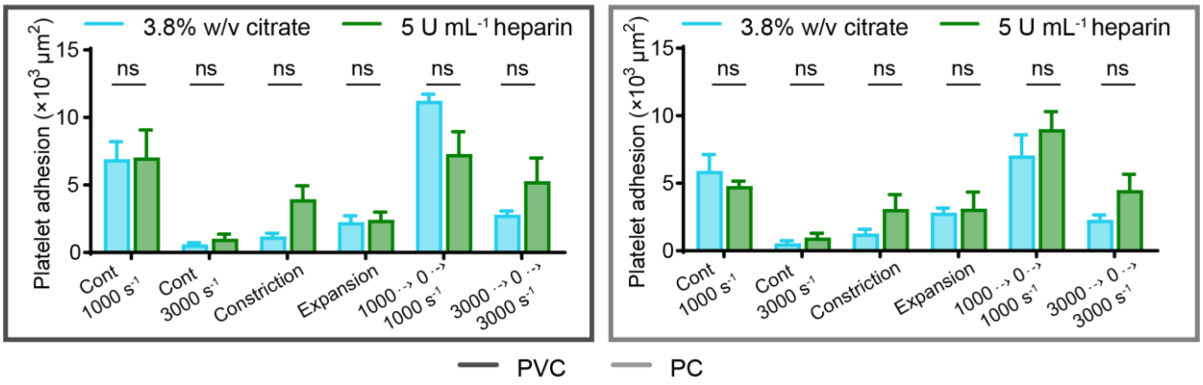

Figure S2. Analysis of total platelet adhesion on PVC and PC at t = 10 min, using either 3.8% w/v citrate or 5 U mL^{-1} heparin showed no statistical difference at all screened flow conditions. Error bars are mean ± SEM, n = 5 donors. Significance comparisons by a two-way ANOVA with Bonferroni's post hoc multiple comparisons test. $P < 0.05$ was considered statistically significant.

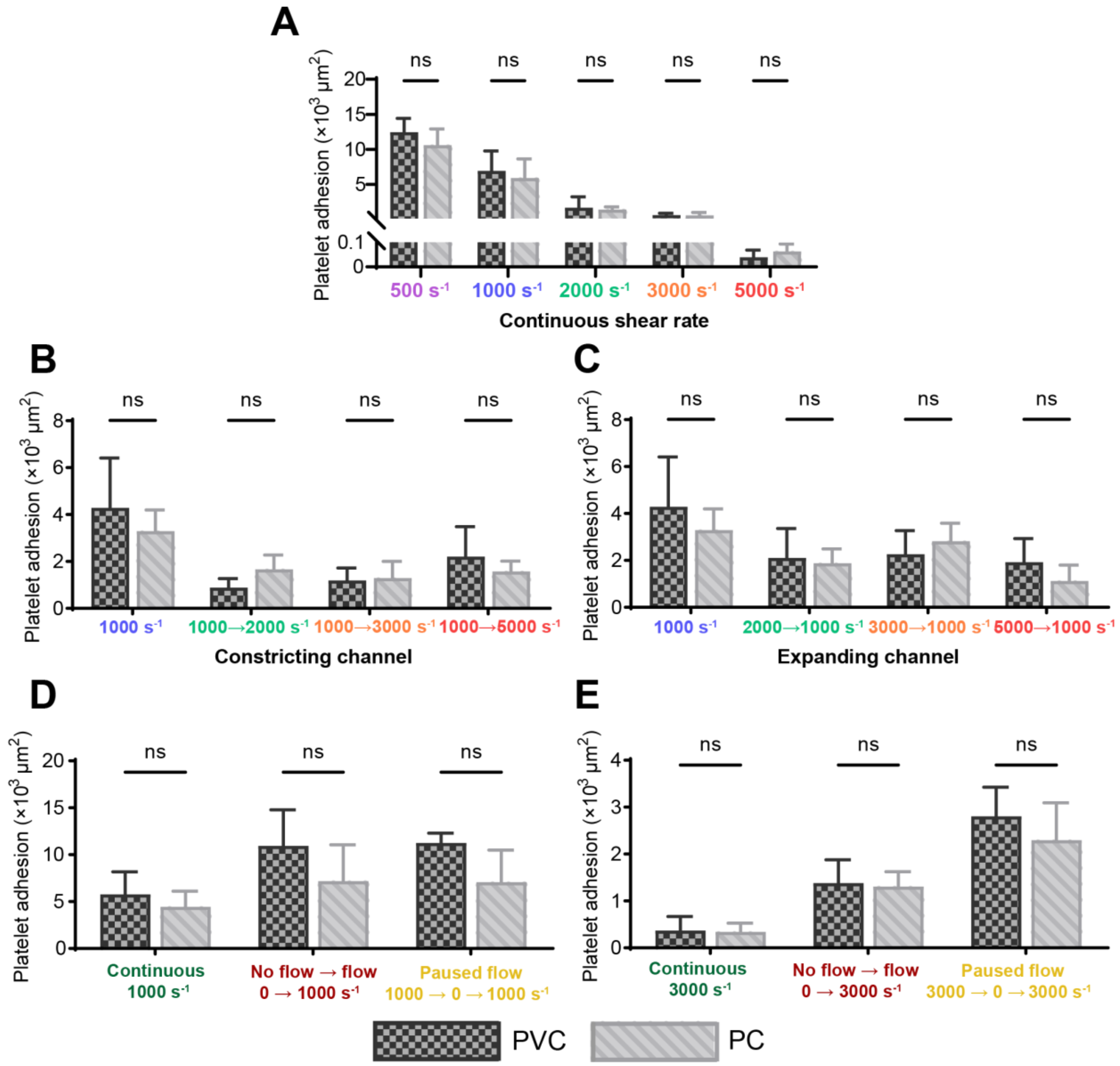

Figure S3. Comparison of platelet adhesion on PVC and PC showed no significant difference. Analysis of total fluorescent surface area indicating platelet adhesion at 10 minutes on PVC vs. PC for **(A)** continuous shear at 500, 1000, 2000, 3000 and 5000 s^{-1}, **(B)** continuous 1000 s^{-1} versus constricting channels at 1000 to 2000 s^{-1}, 1000 to 3000 s^{-1} and 1000 to 5000 s^{-1} conditions **(C)** continuous 1000 s^{-1} versus expanding channels at 2000 to 1000 s^{-1}, 3000 to1000 s^{-1} and 5000 to 1000 s^{-1} conditions **(D)** continuous 1000 s^{-1}, no flow (0 to 1000 s^{-1}) and paused flow (1000 to 0 to 1000 s^{-1}), **(E)** continuous 1000 s^{-1}, no flow (0 to 3000 s^{-1}) and paused flow (3000 to 0 to 3000 s^{-1}). Error bars are mean ± SD, n = 5 donors. Significance comparisons by a two-way ANOVA with Bonferroni's post hoc test. $P < 0.05$ was considered statistically significant.

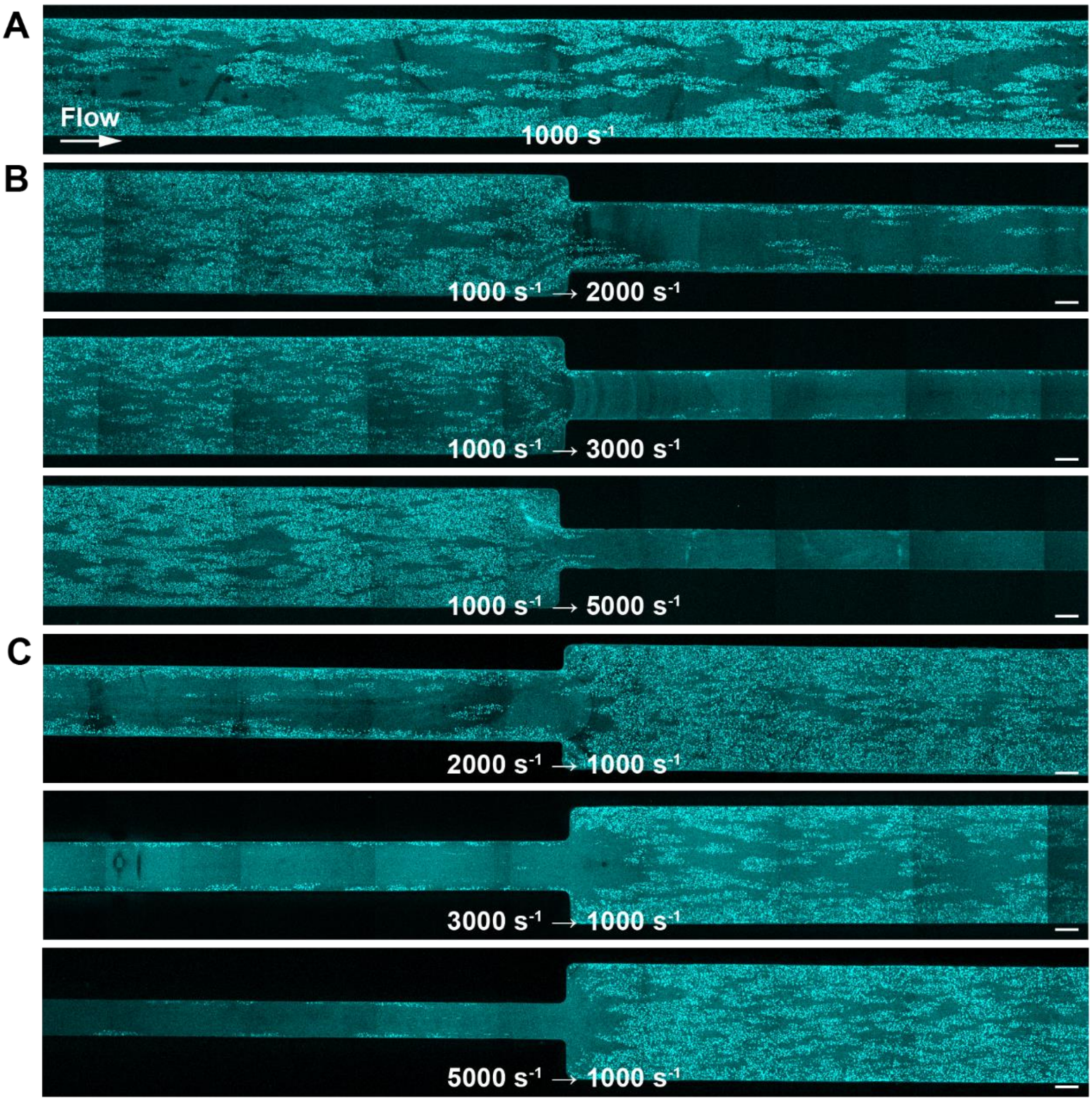

Figure S4. Platelet adhesion at accelerating flow constriction and decelerating flow expansion on PC. Representative confocal micrographs of platelet adhesion (cyan) at t = 10 min, at **(A)** continuous (straight) flow, **(B)** constriction from 1000 s^{-1} to 2000, 3000 and 5000 s^{-1}, and **(C)** expansion from 2000, 3000 and 5000 s^{-1} to 1000 s^{-1}. Scale bar = 50 µm.

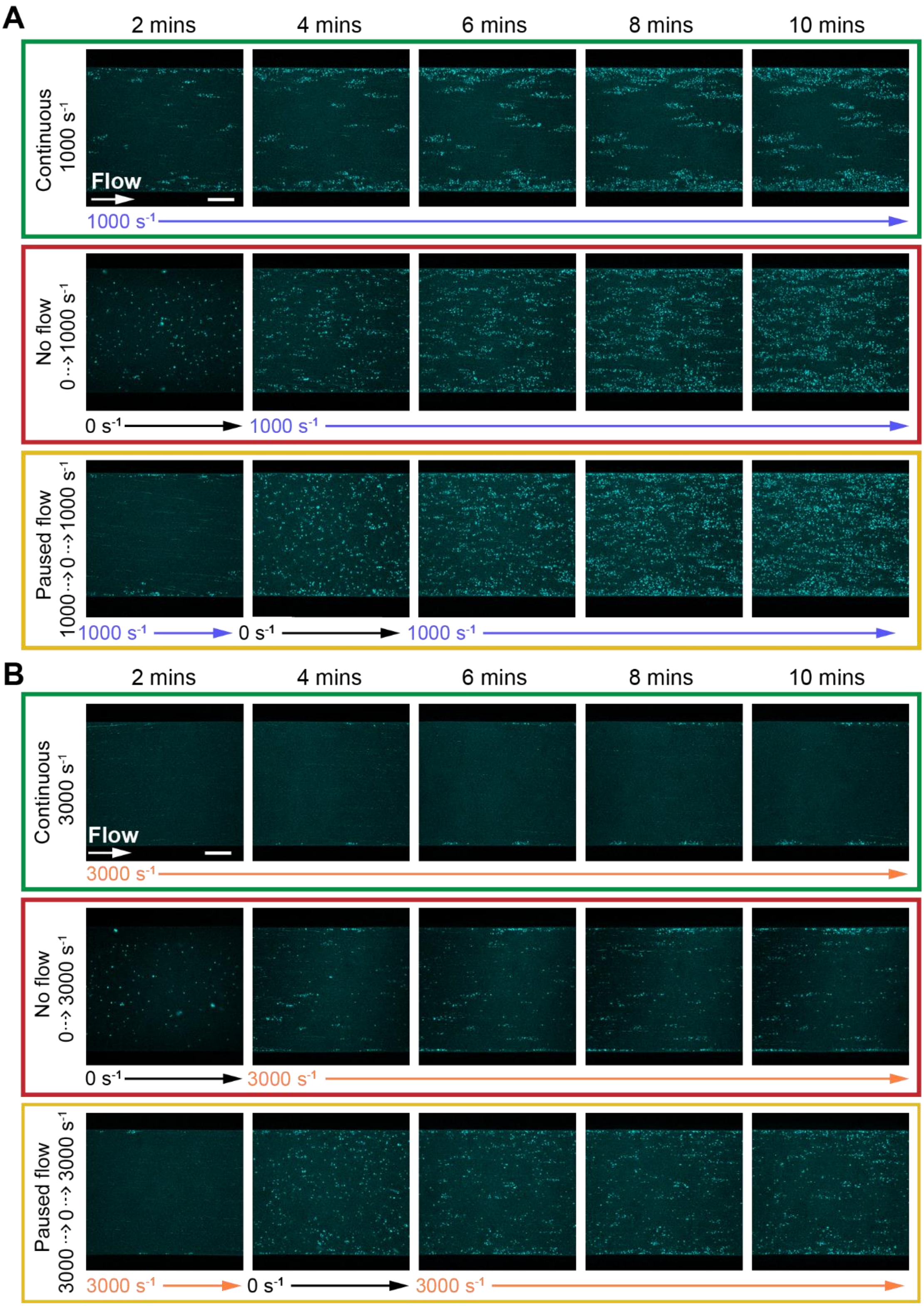

Figure S5. Platelet adhesion at no flow and paused flow conditions on PC. Representative confocal micrographs of platelet adhesion (cyan) on PC over 10 minutes of perfusion for **(A)** 1000 s^{-1}, 0 to 1000 s^{-1} and 1000 to 0 to 1000 s^{-1} flow conditions and **(B)** 3000 s^{-1}, 0 to 3000 s^{-1} and 3000 to 0 to 3000 s^{-1} flow conditions. Scale bar = 50 µm.

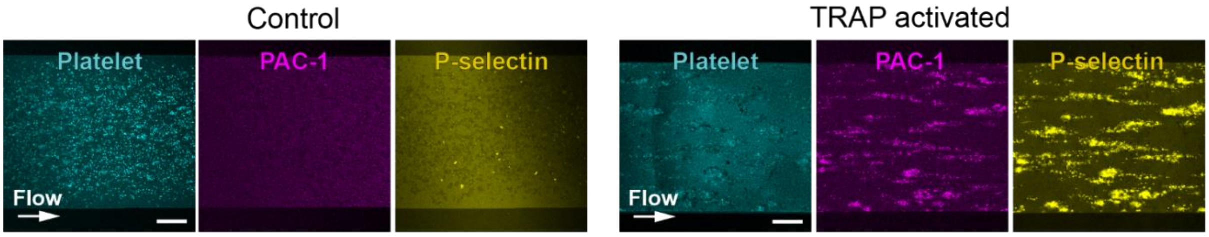

Figure S6. Representative confocal micrographs of control (without thrombin receptor activating peptide (TRAP) activation) platelets compared to TRAP (10 µM) activated platelets, after 2 minutes of flow at 1000 s^{-1}. TRAP activated platelets showed platelet aggregation, and high levels of PAC-1 and P-selectin expression. Scale bar = 50 µm.

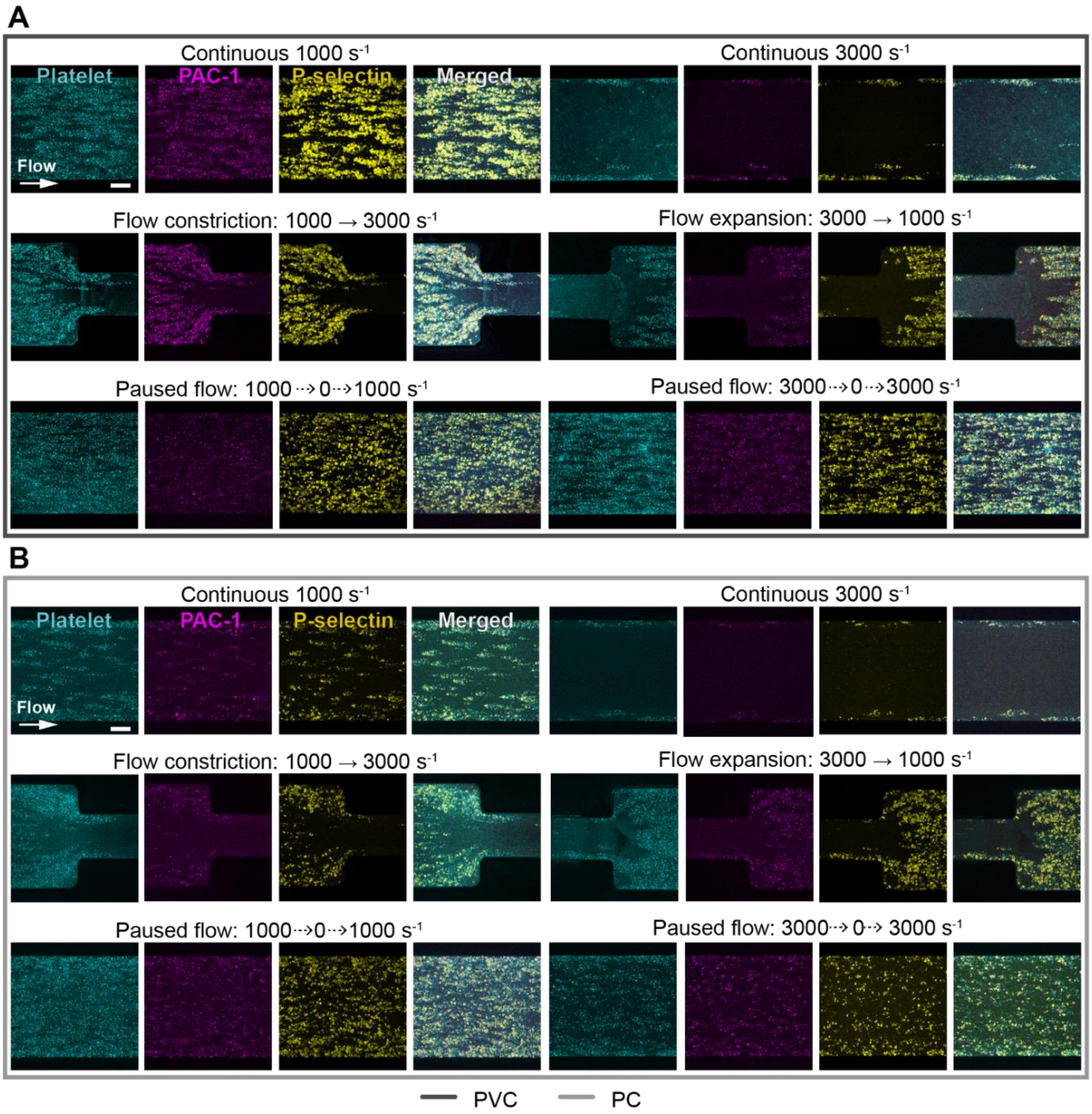

Figure S7. Representative confocal micrographs of platelet adhesion (cyan), PAC-1 (magenta) and P-selectin (yellow) on **(A)** PVC and **(B)** PC after 10 minutes, at various flow conditions. Scale bar = 50 µm.

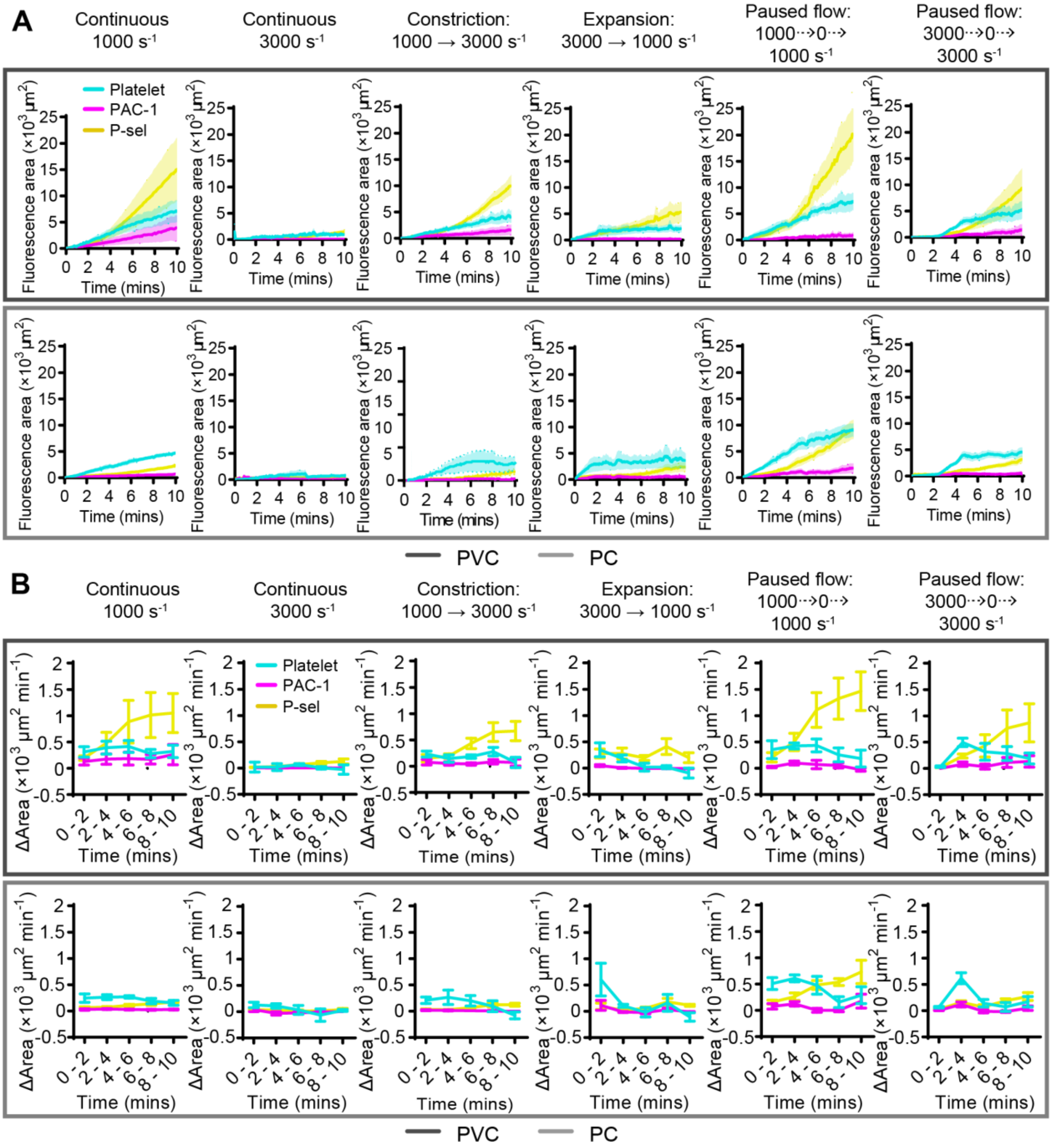

Figure S8. (A) Total fluorescent surface area indicating platelet adhesion (cyan), PAC-1 (magenta) and P-selectin (yellow) expression over 10 minutes on PVC (top row) and PC (bottom row) at various flow conditions. **(B)** Platelet adhesion (cyan), PAC-1 (magenta) and P-selectin (yellow) expression rate over 2-minute periods on PVC (top row) and PC (bottom row) at various flow conditions. Error bars are mean ± SEM, n = 5 donors.
